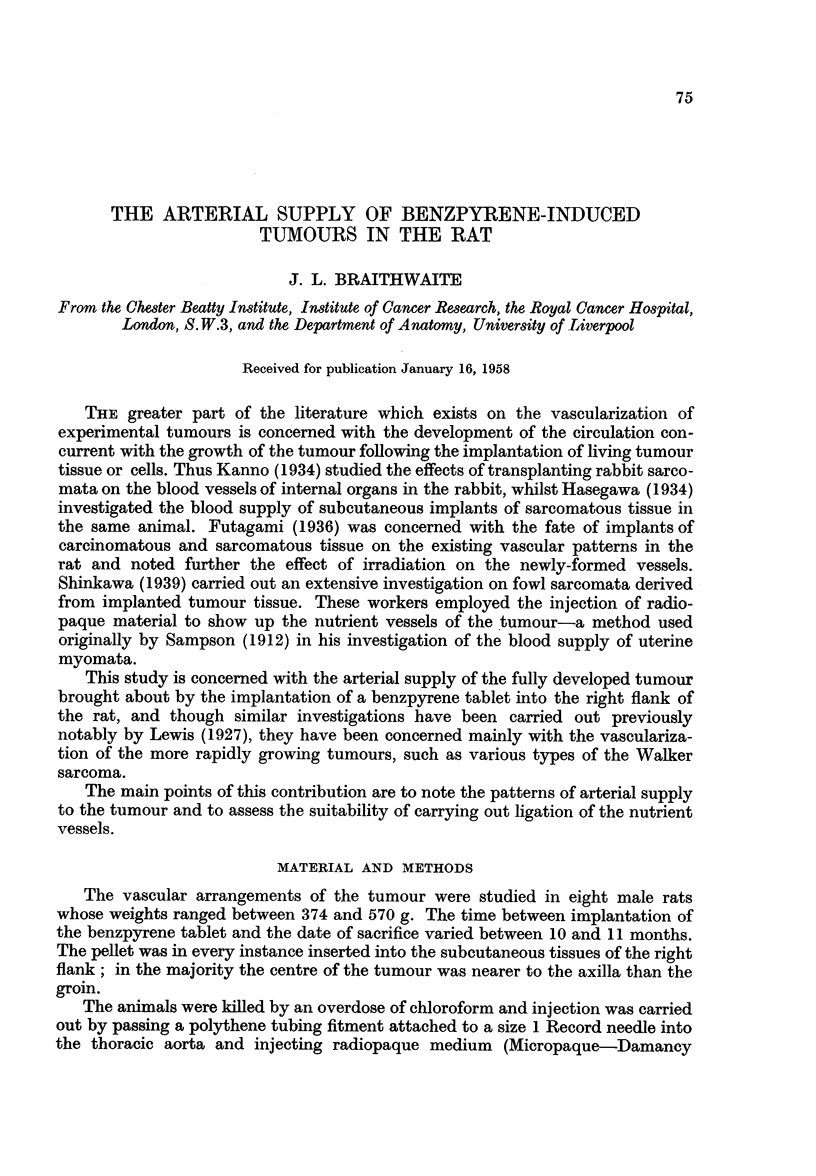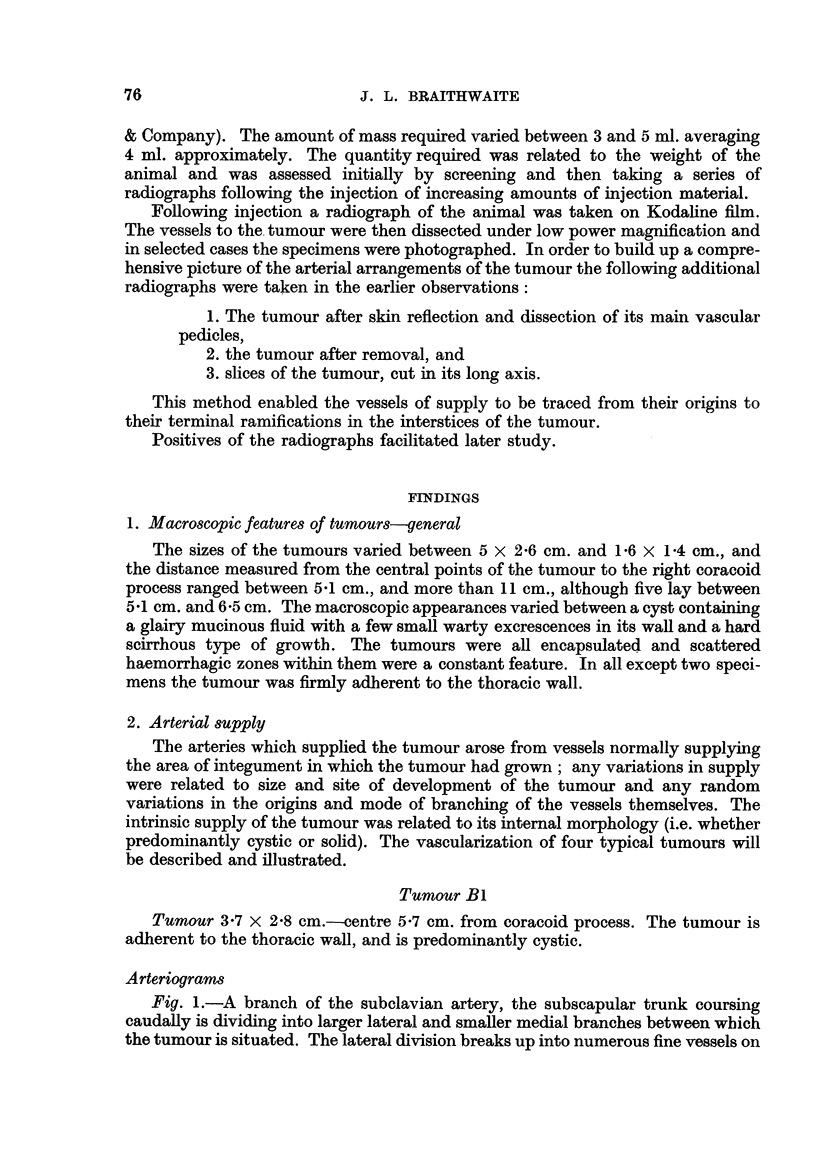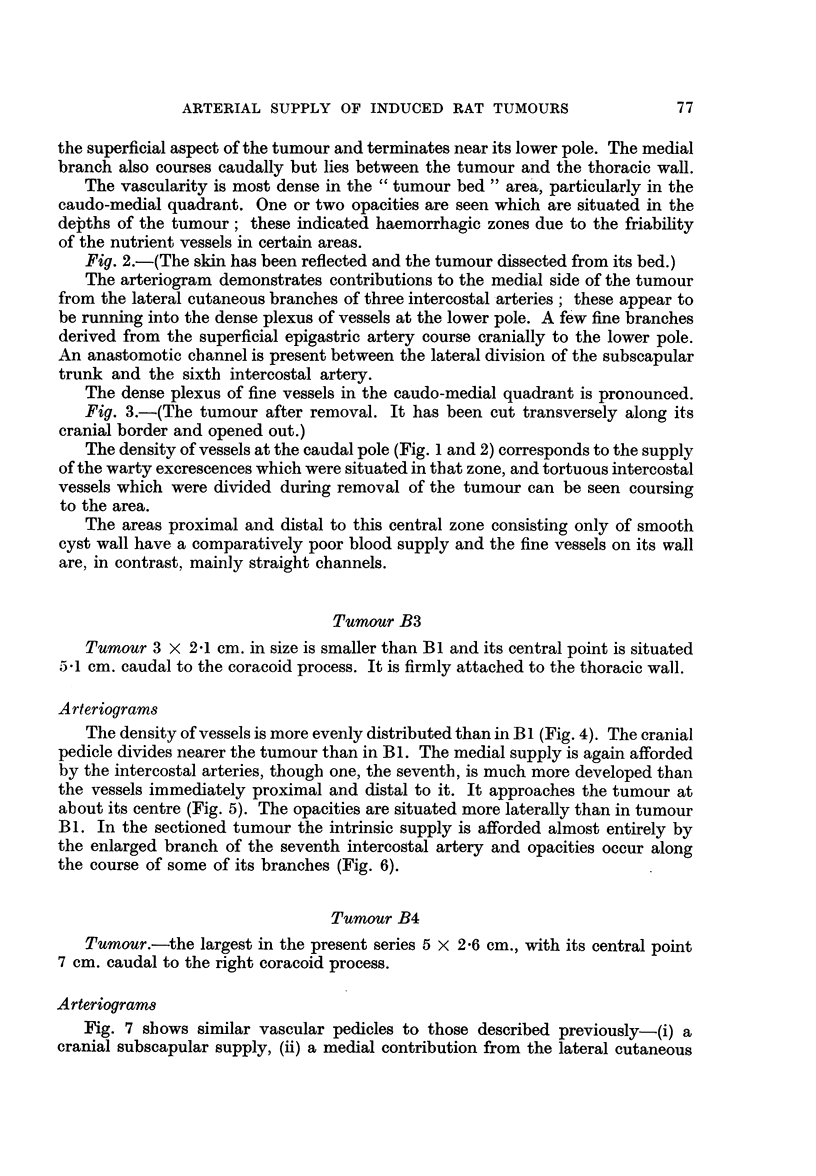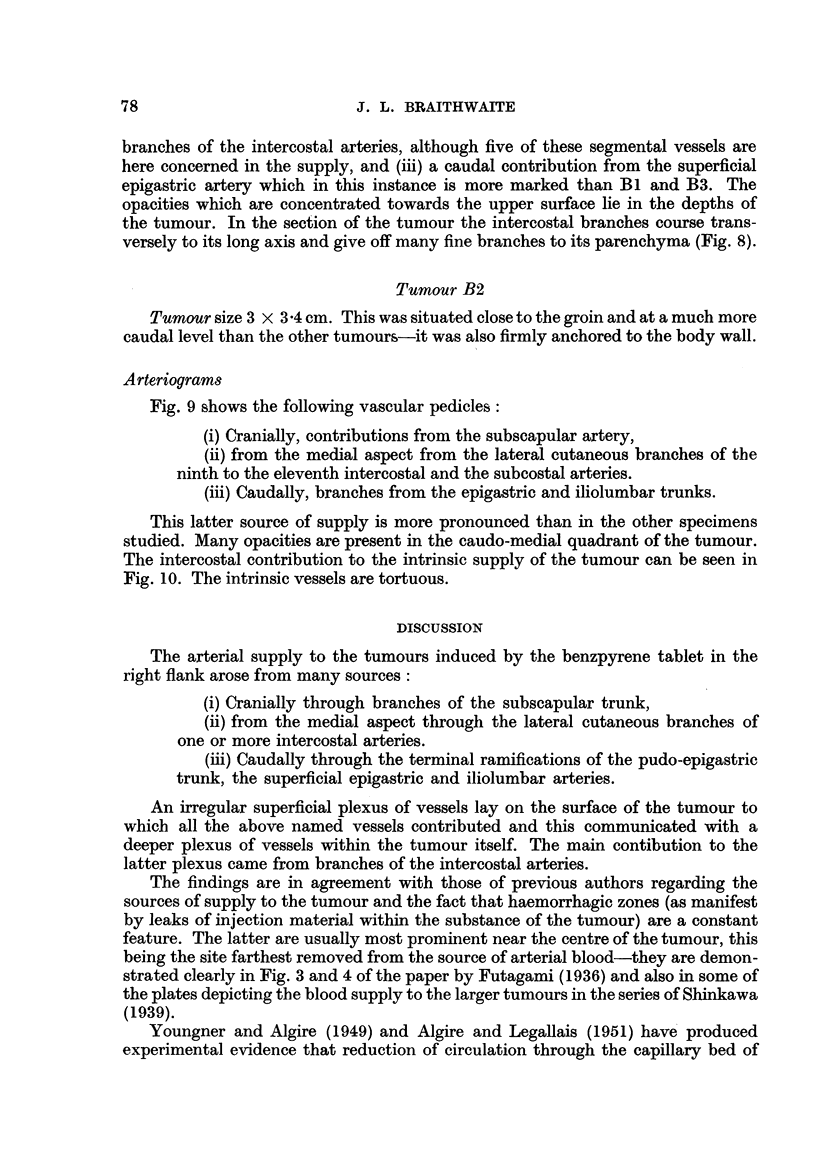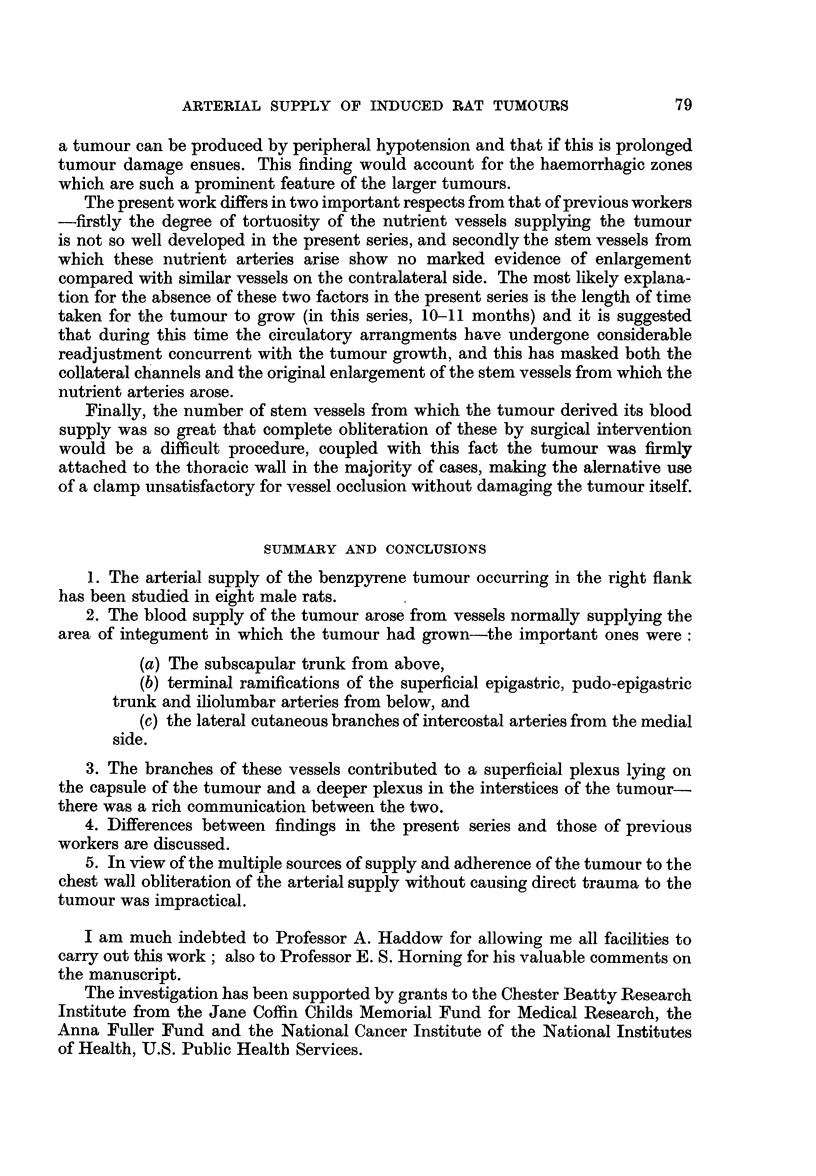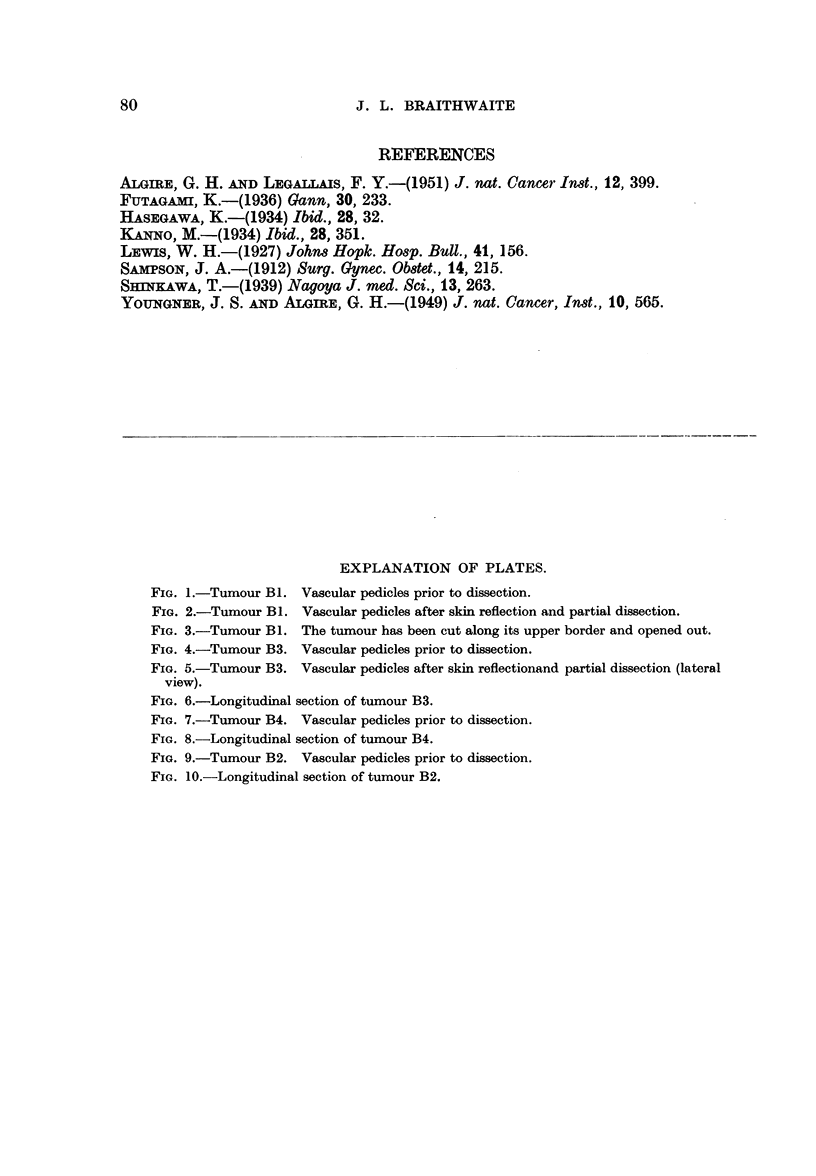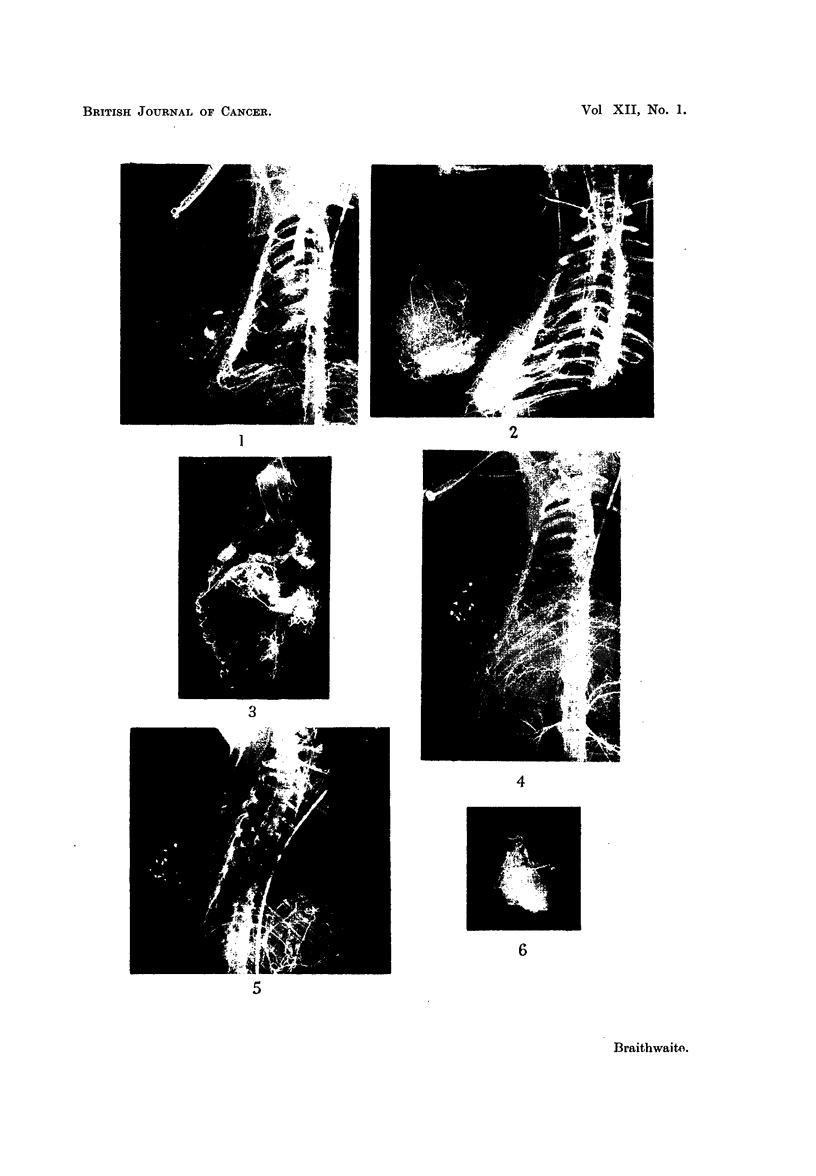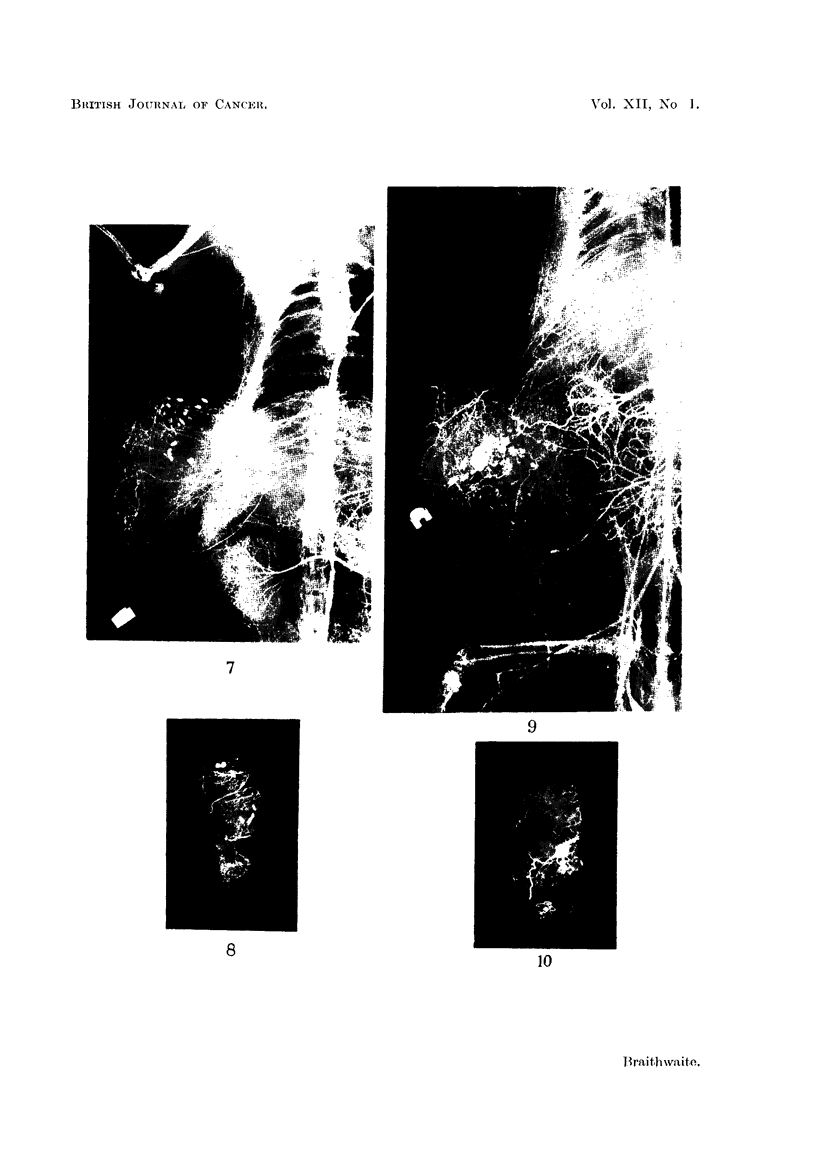# The Arterial Supply of Benzpyrene-Induced Tumours in the Rat

**DOI:** 10.1038/bjc.1958.11

**Published:** 1958-03

**Authors:** J. L. Braithwaite

## Abstract

**Images:**


### THE ARTERIAL SUPPLY OF BENZPYRENE-INDUCED TUMOURS IN THE RAT

J. L. BRAITHWAITE

*From the Chester Beatty Institute, Institute of Cancer Research, the Royal Cancer Hospital, London, S.W.3, and the Department of Anatomy, University of Liverpool*

Received for publication January 16, 1958

THE greater part of the literature which exists on the vascularization of experimental tumours is concerned with the development of the circulation concurrent with the growth of the tumour following the implantation of living tumour tissue or cells. Thus Kanno (1934) studied the effects of transplanting rabbit sarcomata on the blood vessels of internal organs in the rabbit, whilst Hasegawa (1934) investigated the blood supply of subcutaneous implants of sarcomatous tissue in the same animal. Futagami (1936) was concerned with the fate of implants of carcinomatous and sarcomatous tissue on the existing vascular patterns in the rat and noted further the effect of irradiation on the newly-formed vessels. Shinkawa (1939) carried out an extensive investigation on fowl sarcomata derived from implanted tumour tissue. These workers employed the injection of radiopaque material to show up the nutrient vessels of the tumour—a method used originally by Sampson (1912) in his investigation of the blood supply of uterine myomata.

This study is concerned with the arterial supply of the fully developed tumour brought about by the implantation of a benzpyrene tablet into the right flank of the rat, and though similar investigations have been carried out previously notably by Lewis (1927), they have been concerned mainly with the vascularization of the more rapidly growing tumours, such as various types of the Walker sarcoma.

The main points of this contribution are to note the patterns of arterial supply to the tumour and to assess the suitability of carrying out ligation of the nutrient vessels.

### MATERIAL AND METHODS

The vascular arrangements of the tumour were studied in eight male rats whose weights ranged between 374 and 570 g. The time between implantation of the benzpyrene tablet and the date of sacrifice varied between 10 and 11 months. The pellet was in every instance inserted into the subcutaneous tissues of the right flank ; in the majority the centre of the tumour was nearer to the axilla than the groin.

The animals were killed by an overdose of chloroform and injection was carried out by passing a polythene tubing fitment attached to a size 1 Record needle into the thoracic aorta and injecting radiopaque medium (Micropaque—Damancy

& Company). The amount of mass required varied between 3 and 5 ml. averaging 4 ml. approximately. The quantity required was related to the weight of the animal and was assessed initially by screening and then taking a series of radiographs following the injection of increasing amounts of injection material.

Following injection a radiograph of the animal was taken on Kodaline film. The vessels to the tumour were then dissected under low power magnification and in selected cases the specimens were photographed. In order to build up a comprehensive picture of the arterial arrangements of the tumour the following additional radiographs were taken in the earlier observations :

1. The tumour after skin reflection and dissection of its main vascular pedicles,
2. the tumour after removal, and
3. slices of the tumour, cut in its long axis.

This method enabled the vessels of supply to be traced from their origins to their terminal ramifications in the interstices of the tumour.

Positives of the radiographs facilitated later study.

### FINDINGS

### 1. *Macroscopic features of tumours—general*

The sizes of the tumours varied between 5 × 2·6 cm. and 1·6 × 1·4 cm., and the distance measured from the central points of the tumour to the right coracoid process ranged between 5·1 cm., and more than 11 cm., although five lay between 5·1 cm. and 6·5 cm. The macroscopic appearances varied between a cyst containing a glairy mucinous fluid with a few small warty excrescences in its wall and a hard scirrhous type of growth. The tumours were all encapsulated and scattered haemorrhagic zones within them were a constant feature. In all except two specimens the tumour was firmly adherent to the thoracic wall.

### 2. *Arterial supply*

The arteries which supplied the tumour arose from vessels normally supplying the area of integument in which the tumour had grown ; any variations in supply were related to size and site of development of the tumour and any random variations in the origins and mode of branching of the vessels themselves. The intrinsic supply of the tumour was related to its internal morphology (i.e. whether predominantly cystic or solid). The vascularization of four typical tumours will be described and illustrated.

#### *Tumour B*1

*Tumour* 3·7 × 2·8 cm.—centre 5·7 cm. from coracoid process. The tumour is adherent to the thoracic wall, and is predominantly cystic.

#### *Arteriograms*

*Fig.* 1.—A branch of the subclavian artery, the subscapular trunk coursing caudally is dividing into larger lateral and smaller medial branches between which the tumour is situated. The lateral division breaks up into numerous fine vessels on the superficial aspect of the tumour and terminates near its lower pole. The medial branch also courses caudally but lies between the tumour and the thoracic wall.

The vascularity is most dense in the " tumour bed " area, particularly in the caudo-medial quadrant. One or two opacities are seen which are situated in the depths of the tumour ; these indicated haemorrhagic zones due to the friability of the nutrient vessels in certain areas.

*Fig.* 2.—(The skin has been reflected and the tumour dissected from its bed.)

The arteriogram demonstrates contributions to the medial side of the tumour from the lateral cutaneous branches of three intercostal arteries ; these appear to be running into the dense plexus of vessels at the lower pole. A few fine branches derived from the superficial epigastric artery course cranially to the lower pole. An anastomotic channel is present between the lateral division of the subscapular trunk and the sixth intercostal artery.

The dense plexus of fine vessels in the caudo-medial quadrant is pronounced.

*Fig.* 3.—(The tumour after removal. It has been cut transversely along its cranial border and opened out.)

The density of vessels at the caudal pole (Fig. 1 and 2) corresponds to the supply of the warty excrescences which were situated in that zone, and tortuous intercostal vessels which were divided during removal of the tumour can be seen coursing to the area.

The areas proximal and distal to this central zone consisting only of smooth cyst wall have a comparatively poor blood supply and the fine vessels on its wall are, in contrast, mainly straight channels.

### *Tumour B*3

*Tumour* 3 × 2·1 cm. in size is smaller than B1 and its central point is situated 5·1 cm. caudal to the coracoid process. It is firmly attached to the thoracic wall.

### *Arteriograms*

The density of vessels is more evenly distributed than in B1 (Fig. 4). The cranial pedicle divides nearer the tumour than in B1. The medial supply is again afforded by the intercostal arteries, though one, the seventh, is much more developed than the vessels immediately proximal and distal to it. It approaches the tumour at about its centre (Fig. 5). The opacities are situated more laterally than in tumour B1. In the sectioned tumour the intrinsic supply is afforded almost entirely by the enlarged branch of the seventh intercostal artery and opacities occur along the course of some of its branches (Fig. 6).

### *Tumour B*4

*Tumour.*—the largest in the present series 5 × 2·6 cm., with its central point 7 cm. caudal to the right coracoid process.

### *Arteriograms*

Fig. 7 shows similar vascular pedicles to those described previously—(i) a cranial subscapular supply, (ii) a medial contribution from the lateral cutaneous branches of the intercostal arteries, although five of these segmental vessels are here concerned in the supply, and (iii) a caudal contribution from the superficial epigastric artery which in this instance is more marked than B1 and B3. The opacities which are concentrated towards the upper surface lie in the depths of the tumour. In the section of the tumour the intercostal branches course transversely to its long axis and give off many fine branches to its parenchyma (Fig. 8).

### *Tumour B2*

*Tumour* size 3 × 3·4 cm. This was situated close to the groin and at a much more caudal level than the other tumours—it was also firmly anchored to the body wall.

*Arteriograms*

Fig. 9 shows the following vascular pedicles :

(i) Cranially, contributions from the subscapular artery,
(ii) from the medial aspect from the lateral cutaneous branches of the ninth to the eleventh intercostal and the subcostal arteries.
(iii) Caudally, branches from the epigastric and iliolumbar trunks.

This latter source of supply is more pronounced than in the other specimens studied. Many opacities are present in the caudo-medial quadrant of the tumour. The intercostal contribution to the intrinsic supply of the tumour can be seen in Fig. 10. The intrinsic vessels are tortuous.

### DISCUSSION

The arterial supply to the tumours induced by the benzpyrene tablet in the right flank arose from many sources :

(i) Cranially through branches of the subscapular trunk,
(ii) from the medial aspect through the lateral cutaneous branches of one or more intercostal arteries.
(iii) Caudally through the terminal ramifications of the pudo-epigastric trunk, the superficial epigastric and iliolumbar arteries.

An irregular superficial plexus of vessels lay on the surface of the tumour to which all the above named vessels contributed and this communicated with a deeper plexus of vessels within the tumour itself. The main contibution to the latter plexus came from branches of the intercostal arteries.

The findings are in agreement with those of previous authors regarding the sources of supply to the tumour and the fact that haemorrhagic zones (as manifest by leaks of injection material within the substance of the tumour) are a constant feature. The latter are usually most prominent near the centre of the tumour, this being the site farthest removed from the source of arterial blood—they are demonstrated clearly in Fig. 3 and 4 of the paper by Futagami (1936) and also in some of the plates depicting the blood supply to the larger tumours in the series of Shinkawa (1939).

Youngner and Algire (1949) and Algire and Legallais (1951) have produced experimental evidence that reduction of circulation through the capillary bed of a tumour can be produced by peripheral hypotension and that if this is prolonged tumour damage ensues. This finding would account for the haemorrhagic zones which are such a prominent feature of the larger tumours.

The present work differs in two important respects from that of previous workers —firstly the degree of tortuosity of the nutrient vessels supplying the tumour is not so well developed in the present series, and secondly the stem vessels from which these nutrient arteries arise show no marked evidence of enlargement compared with similar vessels on the contralateral side. The most likely explanation for the absence of these two factors in the present series is the length of time taken for the tumour to grow (in this series, 10–11 months) and it is suggested that during this time the circulatory arrangments have undergone considerable readjustment concurrent with the tumour growth, and this has masked both the collateral channels and the original enlargement of the stem vessels from which the nutrient arteries arose.

Finally, the number of stem vessels from which the tumour derived its blood supply was so great that complete obliteration of these by surgical intervention would be a difficult procedure, coupled with this fact the tumour was firmly attached to the thoracic wall in the majority of cases, making the alernative use of a clamp unsatisfactory for vessel occlusion without damaging the tumour itself.

### SUMMARY AND CONCLUSIONS

1. The arterial supply of the benzpyrene tumour occurring in the right flank has been studied in eight male rats.

2. The blood supply of the tumour arose from vessels normally supplying the area of integument in which the tumour had grown—the important ones were :

(*a*) The subscapular trunk from above,

(*b*) terminal ramifications of the superficial epigastric, pudo-epigastric trunk and iliolumbar arteries from below, and

(*c*) the lateral cutaneous branches of intercostal arteries from the medial side.

3. The branches of these vessels contributed to a superficial plexus lying on the capsule of the tumour and a deeper plexus in the interstices of the tumour—there was a rich communication between the two.

4. Differences between findings in the present series and those of previous workers are discussed.

5. In view of the multiple sources of supply and adherence of the tumour to the chest wall obliteration of the arterial supply without causing direct trauma to the tumour was impractical.

I am much indebted to Professor A. Haddow for allowing me all facilities to carry out this work ; also to Professor E. S. Horning for his valuable comments on the manuscript.

The investigation has been supported by grants to the Chester Beatty Research Institute from the Jane Coffin Childs Memorial Fund for Medical Research, the Anna Fuller Fund and the National Cancer Institute of the National Institutes of Health, U.S. Public Health Services.

### REFERENCES

ALGIRE, G. H. AND LEGALLAIS, F. Y.—(1951) *J. nat. Cancer Inst.*, **12**, 399.
FUTAGAMI, K.—(1936) *Gann*, **30**, 233.
HASEGAWA, K.—(1934) *Ibid.*, **28**, 32.
KANNO, M.—(1934) *Ibid.*, **28**, 351.
LEWIS, W. H.—(1927) *Johns Hopk. Hosp. Bull.*, **41**, 156.
SAMPSON, J. A.—(1912) *Surg. Gynec. Obstet.*, **14**, 215.
SHINKAWA, T.—(1939) *Nagoya J. med. Sci.*, **13**, 263.
YOUNGNER, J. S. AND ALGIRE, G. H.—(1949) *J. nat. Cancer, Inst.*, **10**, 565.

---

### EXPLANATION OF PLATES.

FIG. 1.—Tumour B1. Vascular pedicles prior to dissection.

FIG. 2.—Tumour B1. Vascular pedicles after skin reflection and partial dissection.

FIG. 3.—Tumour B1. The tumour has been cut along its upper border and opened out.

FIG. 4.—Tumour B3. Vascular pedicles prior to dissection.

FIG. 5.—Tumour B3. Vascular pedicles after skin reflectionand partial dissection (lateral view).

FIG. 6.—Longitudinal section of tumour B3.

FIG. 7.—Tumour B4. Vascular pedicles prior to dissection.

FIG. 8.—Longitudinal section of tumour B4.

FIG. 9.—Tumour B2. Vascular pedicles prior to dissection.

FIG. 10.—Longitudinal section of tumour B2.